# Hemodialysis vascular access options in pediatrics: considerations for patients and practitioners

**DOI:** 10.1007/s00467-008-0812-3

**Published:** 2009-06-01

**Authors:** Deepa H. Chand, Rudolph P. Valentini, Elaine S. Kamil

**Affiliations:** 1 Pediatric Nephrology and Hypertension, Akron Children’s Hospital, 1 Perkins Square, Akron, OH 44308 USA; 2 The Carman and Ann Adams Department of Pediatrics, Division of Nephrology, Wayne State University School of Medicine, Children’s Hospital of Michigan, Detroit, MI USA; 3 Department of Pediatrics, Cedars-Sinai Medical Center, David Geffen School of Medicine at UCLA, Los Angeles, CA USA

**Keywords:** Pediatric nephrology, Hemodialysis, Arteriovenous fistula, Central venous catheter, Arteriovenous graft

## Abstract

Recent data indicate that the incidence of end-stage renal disease (ESRD) in pediatric patients (age 0–19 years) has increased over the past two decades. Similarly, the prevalence of ESRD has increased threefold over the same period. Hemodialysis (HD) continues to be the most frequently utilized modality for renal replacement therapy in incident pediatric ESRD patients. The number of children on HD exceeded the sum total of those on peritoneal dialysis and those undergoing pre-emptive renal transplantation. Choosing the best vascular access option for pediatric HD patients remains challenging. Despite a national initiative for fistula first in the adult hemodialysis population, the pediatric nephrology community in the United States of America utilizes central venous catheters as the primary dialysis access for most patients. Vascular access management requires proper advance planning to assure that the best permanent access is placed, seamless communication involving a multidisciplinary team of nephrologists, nurses, surgeons, and interventional radiologists, and ongoing monitoring to ensure a long life of use. It is imperative that practitioners have a long-term vision to decrease morbidity in this unique patient population. This article reviews the various types of pediatric vascular accesses used worldwide and the benefits and disadvantages of these various forms of access.

## Introduction

The 2006 report of the United States Renal Data Systems (USRDS) indicated that the incidence of end-stage renal disease (ESRD) in pediatric patients (age 0–19 years) had increased from 8.6 cases per million population in 1980 to 14.1 cases per million population in 2004 [1]. Similarly, the prevalence of ESRD had increased from 28.3 to 81.1 cases per million population over the same period. Hemodialysis (HD) was the most frequently utilized modality for renal replacement therapy in the 1,346 incident pediatric ESRD cases in 2004. The 51% of children on HD exceeded those on peritoneal dialysis (33%) and those undergoing pre-emptive renal transplantation (16%). With regard to prevalence, 68% of the 7,218 children with ESRD had a functioning renal transplant. Of the remaining children (2,311 patients) being treated with dialysis, the majority (59%) were treated with hemodialysis [1].

“The ideal vascular access delivers a flow rate adequate for the dialysis prescription, has a long use-life, and has a low rate of complications (e.g. infection, stenosis, thrombosis, aneurysm, and limb ischemia)” [2]. The arteriovenous fistula (AVF) best approximates this definition. Unfortunately, the access used most often in children in the USA is a central venous catheter (CVC) as opposed to an AVF or arteriovenous graft (AVG). Despite the increasing focus on the potential morbidity associated with CVCs, their usage rate at hemodialysis initiation has increased in recent years, with usage rates of 89% for children < 13 years of age and 64% in those 13–19 years of age [1]. Review of the 2006 Annual report of the North American Pediatric Renal Trials and Collaborative Studies (NAPRTCS) reveals that the majority of pediatric patients (78.9%) receiving hemodialysis have a CVC as primary access, in comparison with AVF (12.3%) and AVG (8.5%) [3]. Dialysis termination in HD patients is most often due to transplantation or patient choice. Specifically, patients with CVCs were more likely to terminate hemodialysis within the first 6 months of dialysis initiation. This would suggest that practitioners are more likely to initiate hemodialysis with a CVC in patients anticipated to receive transplants within a short time. Despite the anticipation of shortened time to transplantation, USRDS data show that, in pediatric patients, the time to first transplantation is actually increasing [1]. From 1996–2000, 75% of children under 11 years of age, and 90.3% of those 11 years of age or older, received a transplant within 5 years of initiation; between 2001 and 2005, the percents were 70 and 79, respectively. Owing to the variability in access use and times to transplantation, the decision of which vascular access type is to be used becomes even more of a conundrum.

Thus, it can be expected that practitioners will need to focus increasing efforts toward optimizing care of the pediatric hemodialysis patient. This is based on the trends of increasing incident and prevalent rates for children with ESRD, accompanied by the fact that HD is the most commonly used modality in incident patients and prevalent patients requiring dialysis. Clearly, choosing the best vascular access option in the pediatric hemodialysis population remains challenging. Despite a national initiative for fistula first in the adult hemodialysis population, to date the pediatric nephrology community in the USA utilizes the CVC as the primary dialysis access in a majority of patients. Although AVFs should be generally preferred in most patients, CVC use may be appropriate in certain settings, including those patients expected to receive a renal transplant within a short time and exceptionally small children (weight < 10 kg). Other factors to be considered include the rate of progression of chronic kidney disease or the patient’s desire to transfer to peritoneal dialysis. This article will attempt to review the various types of pediatric vascular accesses used around the world and the benefits and disadvantages of the various forms of accesses. When appropriate, we attempt to offer suggestions for optimizing each type of vascular access.

## Arteriovenous fistulae

Historically, the original form of arteriovenous access and the precursor of the modern-day AVF was the “Scribner shunt”. This device, described in 1960, was the original vascular access used to provide long-term hemodialysis for adults. This vascular access consisted of two cannulae: one inserted into the radial artery and the other into the adjacent cephalic vein [4]. Considered to be dependable and reliable, this shunt was still fraught with problems, as 50% were reported to need at least one revision and 31% required multiple revisions [5]. Additionally, the Scribner shunt had an average life span of 113 days, with 40% requiring removal for infection or clotting [6]. Nonetheless, the reliability of this technique, and its subsequent modification for use in children, provided the first form of long-term vascular access for hemodialysis [7].

The first AVF was described by Brescia et al. in 1966 and subsequently has become the ideal vascular access in both adults and children. This is mostly due to its low complication rates and long life span [8, 9]. When AVF was first attempted in children, a 50% immediate failure rate was reported [10]. However, as few as 3 years later, Broyer et al. reported acceptable results in 54% of distal AVFs in children less than 20 kg in weight [11]. Over the years, advances in AVF creation, especially with increased surgical experience, have shown primary failure rates as low as 5%. The preferred sites for AVF placement include, in order, radial artery to cephalic vein (radiocephalic), brachial artery to cephalic vein (brachiocephalic), and brachial artery to basilic vein (brachiobasilic, with or without transposition) [2]. Alternatively, an ulnar artery to basilic vein AVF can be created. A femoral artery to saphenous vein AVF has been described but is rarely used [12]. Although preference is given to the non-dominant forearm, ultimately, vessel size is the most important factor in site selection. If the site with the largest possible venous diameter is chosen, there is greater likelihood of successful use of the AVF. Although definite guidelines regarding minimum vessel size do not exist, general consensus implies a preferred minimum of 2.5 mm venous diameter [13]. Duplex ultrasound scanning by the surgeon, or venography, can provide information regarding adequate vessel size, venous stenosis, or occlusion, and should be considered in pediatric patients so that the best location for AVF placement can be determined [13].

The Centers for Medicare and Medicaid Services (CMS) introduced the “Fistula First” initiative in 2003, with the goal of achieving the guidelines of the National Kidney Foundation/ Kidney Disease Outcomes and Quality Initiative (NKF/K-DOQI). These guidelines recommend a 50% AVF rate in all incident HD patients and a 40% prevalent AVF rate in adult hemodialysis patients. This has not been extrapolated to the pediatric population. However, if the goal is to provide HD with the best possible access, an AVF needs to be considered in all pediatric HD populations as well. It has been the authors’ experience that high AVF usage rates can be established through a multidisciplinary team approach involving pediatric nephrologists, experienced HD nurses, vascular surgeons, interventional radiologists and recreational therapists/child life specialists. Gradman et al. reported the successful creation of 47 AVFs in the USA by utilization of an operating microscope in individuals with small arteries [13]. In fact, a pediatric dialysis unit in Northern Ohio has an average AVF usage rate of > 80%. Microsurgical techniques have been successfully utilized by an experienced vascular surgeon to create AVFs in three children under 15 kg in weight (Chand, unpublished data). Numerous reports of successful creation of AVFs in children have been reported throughout the world, with primary failure rates as low as 5% [14–16]. Time to maturation may be prolonged, with reports of up to 6 months. Thus, early planning should be undertaken to allow time for maturation and/or revision if necessary.

Potential benefits of AVF creation include lower infection rate, lower thrombosis and stenosis rate, and greater freedom with regards to activity. Infection rates for AVFs and AVGs are approximately seven-times lower than those for CVCs. After 6 months of receiving hemodialysis, only 5% of pediatric patients dialyzed via AVFs had developed an access infection, in comparison with 36% of those dialyzed via CVCs [1]. Children with an internal HD access (AVF or AVG) enjoy the luxury of bathing and swimming without concerns of infection risks. Possible complications of AVFs include stenosis, thrombosis, possible leg length discrepancy if the AVF is placed in the lower extremity, and steal syndrome. Steal syndrome occurs when cardiac output is diverted from the capillary bed by the AVF, causing distal ischemia. Additionally, congestive heart failure has been rarely associated with large AVFs in adults, particularly in upper arm fistulae [17].

## Arteriovenous grafts

Arteriovenous grafts (AVG) should be considered as an option for hemodialysis access in children, especially in those who require the replacement of native vessels to perform an adequate anastomosis. Advantages and disadvantages of AVG in comparison with AVF are depicted in Table 1. Alternative materials available include saphenous vein, bovine, umbilical, Dacron, polyurethane, cryopreserved femoral vein, and polytetrafluoroethylene (PTFE). The PTFE graft is the most commonly utilized, due to the fewest complication rates. One study comparing bovine grafts with PTFE grafts demonstrated fewer complications with PTFE ones, specifically fewer thromboses and lower infection rates. Furthermore, the life span of the PTFE graft is comparable to that of its predecessors; the PTFE graft is easier to obtain and easier to repair [18]. Grafts are most commonly placed in the forearm, between the brachial artery and basilic or brachial vein [19]. Alternatively, grafts can be placed between the brachial artery and basilic or axillary vein, just below the axilla. The thigh, with the femoral artery and femoral or saphenous vein being used, is an alternative site often used in small children. Some authors have reported the use of alternative, more creative, AVGs in children in order to optimize care for the long term. Lau et al. reported the combined use of polyurethane and PTFE to create a composite AVG in a child who did not have adequate vessels for an AVF [20]. This AVG was successfully used until the time of transplantation. Of note, higher infection rates have been noted with thigh grafts than with upper extremity grafts [21, 22].

**Table 1:** Permanent vascular access options: AV fistula versus AV graft

AV fistula	AV graft
Lower infection rate	Higher infection rate
Lower thrombosis rate	Higher thrombosis rate
May take 3–6 months to mature	Usually able to be used within a few weeks
Primary failure rate is higher	Primary failure rate is lower
Secondary failure rates lower	Secondary failure rates higher

Advantages for the use of an AVG include a shorter time to first use, higher primary patency rate, and ease of technical creation. Sheth et al. reported the creation of 24 AVFs and 28 AVGs in 19 and 23 pediatric HD patients, respectively. The primary failure rates were 33.3% and 3.6% in AVF and AVG, respectively. The most common site of AVG placement was the thigh, in approximately 50% of patients. Access stenoses and infection were reportedly higher in AVGs than in AVFs, but thrombotic episodes were not significantly different [23]. Chand et al. evaluated dialysis adequacy (Kt/V, urea reduction rates), anemia management, and albumin status based on vascular access type and found no significant differences between AVFs and AVGs [24].

Disadvantages of AVG use include thrombosis, stenosis, and infection. Ramage et al. reported long-term complication rates of AVFs compared with AVG in a retrospective study conducted over a 20-year period. Intervention rates were reported as 17.8% for AVFs and 33% for AVGs. Reasons for discontinuation of AVG use was infection (20%), thrombosis (73%), and planned termination of use (6.7%) [9]. Furthermore, infections are problematic with AVGs and may require graft removal, especially if occurrence is within weeks of placement or results in sepsis. Steal syndrome, as described previously, is a possible complication of AVGs, as well.

## Central venous catheters

Central venous catheters are the most commonly used vascular access in children in North America. A CVC is the first choice of vascular access in the patient who requires urgent hemodialysis, such as the patient who presents in stage V chronic kidney disease (CKD). Alternatively, it can serve as a bridge for a patient who is expected to receive a planned renal transplant or is training to transfer to peritoneal dialysis. There are advantages to a CVC, including the ability for it to be used it immediately and the absence of needle cannulation. However, the disadvantages of using a CVC include its short life span, thrombosis, infection, malfunction and possible fibrin sheath formation [9]. The advantages and disadvantages of CVCs are summarized in Table 2. Median survival times of CVCs have been reported to range from 4 months to 10.6 months [9, 25]. Goldstein et al. evaluated catheter survival times in 56 uncuffed and 22 cuffed CVCs. The median survival period of the uncuffed catheters was only 31 days and of cuffed catheters was 123 days. The 1-year survival rate of long-term use of cuffed catheters was 27% [26]. In many situations, CVC use cannot be avoided and may even be warranted. In those children whose vasculature is too small (particularly children weighing under 10 kg), especially without an experienced surgeon, a CVC may be the best temporary solution. However, periodic reassessments of the vasculature also need to be made. Similarly, if a child has extremity contractures, bony deformities, or other mobility limiting conditions, a CVC may be the best choice to allow nursing personnel ready access.

**Table 2:** Pros and cons of central venous catheters for hemodialysis in children

Pros	Cons
Easily placed	Infection rates high
Can be used immediately	Failure rates and replacement rates high
Painless to the patient	Blood flow rates are variable, leading to potentially poor clearance
Requires little planning prior to placement	Permanent damage to central venous system (stenosis/thrombosis) may occur
Easily removed if used as “transitional” access for future PD or transplant patients	Damage to central vessels can prohibit future AVF/AVG placement in ipsilateral extremity
No vascular steal	Possible Arrhythmia
Decreased risk of high-output cardiac failure	

If a CVC is deemed to be the best option for a patient, good communication between the nephrologist and surgeon/interventional radiologist regarding placement must occur. First, the choice of location is paramount. The NKF/K-DOQI guidelines recommend that the order of CVC placement be sequential: the right internal jugular vein, right external jugular vein, the left internal and external jugular veins, subclavian veins, femoral veins, or translumbar access to the inferior vena cava [2]. This order is based on complication rates from lowest to highest. For example, Schon and Whittman found that the incidence of stenosis was 27% for CVCs placed in the right internal jugular vein in comparison with 40% for those placed in the left internal jugular vein [27]. As such, there must be discussion between the professional placing the CVC and the nephrologist as to the optimal desired location. For example, in order to achieve repeated consistently good blood flow rates, the tip of the CVC should be placed in the right atrium and the line needs to be long enough to be in the appropriate location [28, 29]. When a child moves vigorously, there is potential for the line to move as well. The catheter tip can potentially move out of the right atrium and provide inadequate dialysis. The largest diameter CVC that can safely be placed should be chosen to optimize blood flows as well. Furthermore, kinking of a CVC can be a common problem. Goldstein et al. reported kinking to be the most common reason for central line removal in uncuffed catheters (36%) and second most common reason in cuffed catheters (13.6%) [26]. Kinking can potentially be prevented by bending the catheter in the shape of a C or U prior to tunneling the line. This creates a contiguous arc in the catheter to avoid constriction of the lumen [27].

The most common reason for cuffed CVC removal is infections. These can range from exit site infections to tunnel infections to bacteremia. USRDS data have shown sepsis rates associated with CVCs to approximate 80 per 100 patient-years, as compared to only 10 per 100 patient-years for AVFs [1]. Potential consequences include septic shock, subacute bacterial endocarditis, osteomyelitis and epidural abscess [30, 31]. Infection risk can be decreased by the use of sterile techniques when CVCs are being placed or replaced, by hand washing between dialysis patients (not just changing gloves), and by the use of antibacterial antiseptic solutions for exit site care. One study evaluated potential differences in infection rates based on the use of three agents: 2% chlorhexidine, 10% povidone-iodine, and 70% alcohol, and found that the rates of infection in each group were 0.5%, 2.6%, and 2.3%, respectively [32]. Similarly, bacterial colonization rates were 2.3%, 9.3 and 7.1%, respectively. When various occlusive dressings were assessed, no difference in exit site infection rates was found between transparent dressings and dressings with higher transmissions of moisture vapor. Recently, antimicrobial catheter locks (ACLs) have been used to reduce CVC infection rates. Agents that have previously been used include citrate, aminoglycosides, aminoglycosides + citrate, aminoglycosides + heparin, minocycline + ethylenediamine tetra-acetic acid (EDTA), cephalosporin-based solutions, and vancomycin heparin locks. Gentamicin-based solutions are the least expensive and are most likely the current agents of choice. Concerns of antimicrobial resistance preclude the use of vancomycin in most cases. Given that minocycline is not commercially available, an alternative would be doxycycline EDTA. Studies would suggest that citrate is the ideal ACL solution; however, it is not available in the USA [33].

Thrombus formation is another potential complication of long-term CVC use. In a recent multi-center survey conducted by Valentini et al., a clot was suspected by dialysis providers in 26% of dysfunctional catheters [34]. It can occur in the right atrial wall, on the vessel wall, or may completely occlude the vessel. Tissue ingrowth into the CVC itself has been reported and has entrapped the CVC onto the endothelial surface of the vessel. This, potentially, would require either leaving the CVC in the vessel permanently or thoracotomy for removal [27, 35].

A common problem in long-term CVC use is the formation of a fibrin sheath, which has been reported in up to 50% of CVCs [35]. Over time, the sheath can elongate, covering the intake and outflow holes of the catheter, leading to dysfunction. Of note, if a CVC is replaced into the same fibrin sheath, there will be no improvement in flow, possibly making the vessel unusable. Treatment options including infusion of thrombolytic agents (i.e. tissue plasminogen activator or urokinase) and fibrin sheath stripping, and disruption of the sheath at catheter exchange has been reported, with varying success, high costs, and potential complications [36–38].

For these reasons, the CVC remains a suboptimal choice for hemodialysis vascular access and should be considered as a bridge to a more permanent, optimized, vascular access.

## Caveats regarding placement of access

Early planning is critical in vascular access management. To reiterate, the guidelines of The National Kidney Foundation/ Kidney Disease Outcomes and Quality Initiative (NKF/K-DOQI) recommend a 50% AVF rate in all incident HD patients. Based on a recent study of 37 incident pediatric hemodialysis patients conducted in the Midwestern USA, a majority of patients were followed-up by a nephrologist for at least 6 months, however, a CVC (83.7%) was the predominant vascular access at dialysis initiation [39]. This exemplifies the fact that nephrologists can, and need to, advocate early planning for vascular access placement. At stage IV CKD, the patient who is not anticipated to receive a transplant within 6 months or undergo PD should be referred to an experienced vascular access surgeon, locally or regionally, to be examined for permanent access. Davidson et al. introduced a mantra which is especially applicable to the pediatric HD population: “The issue is not who places the access but who does it right, every time, to everyone, and everywhere” [40]. With a potential barrier being lack of surgical expertise, it is important for the nephrology practitioner to seek an experienced vascular access surgeon, potentially outside one’s own institution, to provide optimal patient care. Choice of center should be outcome and patient driven. Keeping in mind that an AVF can take up to 6 months to mature, early placement should be planned. Furthermore, the patient and family should be educated regarding protection of the non-dominant arm: avoiding venipunctures, blood pressure measurements, etc. In addition, it is important that the placement of peripherally inserted central catheters (PICCs) be avoided. Education regarding cannulation, with the use of a child life specialist, recreational therapist, or peer-to-peer observation/interaction, can be valuable in cannulation preparedness. In essence, a catheter avoidance approach should be undertaken whenever possible.

When one is considering which type of access to place, particular importance should be placed on the preoperative surgical management. A good physical examination includes evaluation of the quality of arterial pulse, to assess for arterial occlusion or impairment of arterial flow. This is particularly true in those patients with a history of peripheral arterial lines. Venous elasticity can be assessed by measurement of venous caliber before and after venostasis is applied, and venous length can be assessed by manual percussion and palpation of the vessel [19]. Elevation of the limb above the head can be used for the assessment of venous drainage. By taking a thorough history and conducting a physical examination, one completes the initial office-based evaluation for AVF creation.

Owing to the small vessels of pediatric patients, radiographic tools can be utilized to assess vascular adequacy better. For example, Gradman et al. reported using bedside duplex ultrasound conducted by the surgeon in person to assess vessel caliber [13]. Others have utilized venography to evaluate vessel length, caliber, and distal and central patency [13, 23].

Intraoperative adjuncts previously reported include loupes, microsutures, and the operating microscope. In fact, some have advocated the use of microsurgical techniques to attain patency rates that parallel those for the adult hemodialysis population [14]. Additional intraoperative adjuncts reported include the use of an inflatable tourniquet to provide maximal exsanguination of the vessel, providing a controlled hemostasis to decrease arterial spasm and microvascular bleeding [14].

If an AVF fails to show signs of maturation within 4–6 weeks of placement, or it develops secondary failure, an evaluation should be conducted. Some have advocated the use of routine ultrasound post-operatively to assure patency and the possibility of deep vein location. In the case of deep location of the vein, the AVF may be patent but inaccessible [41]. As such, surgical transposition to superficialize the vein is most often required. Alternatively, if a stenosis is present, it can be either dilated or surgically revised. If the AVF was placed within 4 weeks, surgery should be considered, as angioplasty may put the anastomosis at risk of rupture. However, if the AVF has been in place for more than 4 weeks, an experienced interventional radiologist should be consulted. Angiography is often performed, and stenosis, if found, may be treated with angioplasty. Again, it is important for the practitioner to be responsive to all access failures and to intervene early so as to optimize the long-term success of the AVF.

## Ongoing monitoring

Once an AVF has been created and successfully used, it is imperative that ongoing maintenance be performed to allow a long life of use. Overall, proactive monitoring of vascular access can decrease frequency of thrombosis and hospitalization. Although monitoring the CVC mostly involves troubleshooting as problems arise, surveillance of AVFs and AVGs is quite plausible. Many times, nursing personnel are the first to detect problems, as exemplified by difficulty in cannulation, poor blood flows, pain, or prolonged bleeding from needle sites. Many techniques for ongoing monitoring have been suggested and include static venous pressure monitoring, dynamic venous pressure monitoring, and ultrasound dilution techniques. Chand et al. reported that dynamic venous pressure monitoring does not adequately predict access failure in pediatric hemodialysis patients when the patients are used as their own historic controls [42]. Although not a randomized controlled trial, it was the only study evaluating venous pressure monitoring in a pediatric patient population to date. Goldstein et al. described the use of an ultrasound dilution technique on a regular basis in pediatric HD patients in order to improve the life of the access [43]. Use of the ultrasound dilution method to monitor the access resulted in a 50% reduction in the number of patients hospitalized, and a significant reduction of costs associated with these hospitalizations. Use of the ultrasound dilution method can decrease morbidity and be a useful adjunct in vascular access management in pediatric patients. This is further supported by the NKF/K-DOQI guidelines for pediatric vascular access [44]. Many times, the ongoing surveillance can be made into a protocol to allow early intervention as warranted. Although there are minor differences between AVF and AVG regarding anatomy and vascular compliance, at this point, no separate pediatric monitoring or intervention guidelines regarding stenosis/thrombosis exist.

Based on review of the current literature, the authors would propose the following as tools for ongoing monitoring of AVFs and AVGs:

1. Inspection: the access should be assessed weekly via inspection, palpation, and auscultation by the nursing staff, with specific attention to arm swelling, prolonged bleeding after needle removal, or change in thrill or bruit. The nephrologist should also inspect the access at each physical examination. Any difficulties in needle cannulation or decreases in blood flow due to elevated negative arterial pump pressures should be noted, as well.
2. Surveillance: decreases in Kt/V or urea reduction ratios should be noted. Determination of access recirculation should be documented on a monthly basis, as well. An adjunct should be used to determine blood flow through the vascular access. If the equipment is available, ultrasound dilution measurements should be performed by a consistent person each month. If such equipment is not available, a Doppler ultrasound can be performed each month.
3. Referral: referral for fistulogram with possible angioplasty should be made if there is (1) inadequate blood flow, thereby compromising adequacy, (2) elevated access recirculation (> 20% after correction of the needle position), (3) corrected access flow less than 650 ml/min per 1.73m^2^ body surface area by ultrasound dilution techniques, (4) consistent abnormality on Doppler ultrasound, or (5) pseudoaneurysm has formed (note: rotation of puncture sites can help minimize risk of pseudoaneurysm formation).

Monthly review of vascular access should be conducted by a multidisciplinary team, ideally involving the nephrologist, nursing staff, and the vascular access surgeon.

## Summary

Vascular access in the pediatric HD patient is a challenging, but necessary, undertaking for the practitioner. It requires proper advance planning to assure that the best permanent access is placed, seamless communication involving a multidisciplinary team of nephrologists, nurses, surgeons, and interventional radiologists, and ongoing monitoring to ensure its long life span. It is imperative that the practitioner have a long-term vision to decrease morbidity in this unique patient population. We recommend an approach to minimize/avoid the prolonged use of CVCs due to risks of infection and the need for vessel preservation. We encourage the practitioner to use good clinical judgment when choosing the appropriate vascular access in each patient.

## **Questions** (Answers appear after the reference list)

1. Which of the following members should *not* be involved as part of a multidisciplinary team approach to successful AVF creation?
  - Experienced hemodialysis nurses
  - Interventional radiologists
  - Pediatric nephrologists
  - Recreational therapists/child life specialists
  - Vascular access surgeons
  - None of the above (all should be involved)
2. All of the following are potential complications of CVC use in pediatric hemodialysis patients except:
  - Infection
  - Malfunction
  - Steal phenomenon
  - Thrombosis
3. Of the following, which is the best method for ongoing surveillance of AVF?
  - Dynamic venous pressure monitoring
  - Static venous pressure monitoring
  - Troubleshooting once a problem arises
  - Ultrasound dilution
4. A 10-year-old boy has a left radiocephalic AVF created, and, 2 weeks after placement, there is no longer a bruit or palpable thrill. Bedside Doppler ultrasound does not show flow through the access. What would be the next step in the management of this patient’s condition?
  - Conduct an evaluation for hypercoagulable state
  - Infuse tissue plasminogen activator through the fistula
  - Refer the patient to interventional radiology
  - Refer the patient to the vascular access surgeon
5. All of the following are factors to be considered in site selection of AVF creation except:
  - Extremity contractures
  - Prior metacarpal fracture
  - Pulse quality
  - Vessel size

## References

[1] US Renal Data System (2006) Annual data report: Atlas of end-stage renal disease in the United States, National Institute of Health, National Institute of Diabetes and Digestive and Kidney Diseases, Bethesda, MD, 2006

[2] National Kidney Foundation-Dialysis Outcomes Quality Initiative (1997). NKF-DOQI clinical practice guidelines for vascular access. Am J Kidney Dis.

[3] North American Pediatric Renal Trials and Collaborative Studies (NAPRTCS) (2006) Annual Report. Dialysis Access data. Available at https://doi.org/web.emmes.com/study/ped/annlrept/annlrept.html 10.1111/j.1399-3046.2007.00704.x 17493215

[4] Quinton W, Dillard D, Scribner BH (1960). Cannulation of blood vessels for prolonged hemodialysis. Trans Am Soc Artif Intern Organs.

[5] Franzone AJ, Tucker BL, Brennan LP, Fine RN, Stiles QR (1971). Hemodialysis in children. Experience with arteriovenous shunts. Arch Surg.

[6] Idriss FS, Nikaidoh H, King LR, Swenson O (1971). Arteriovenous shunts for hemodialysis in infants and children. J Pediatr Surg.

[7] Buselmeier TJ, Kjellstrand CM, Rattazzi LC, Simmons RL, Najarian JS (1972). A new subcutaneous prosthetic A-V shunt: advantages over the standard Quinton-Scribner shunt and A-V fistula. Proc Clin Dial Transplant Forum.

[8] Brescia MJ, Cimino JE, Appel K, Hurwich BJ (1966). Chronic hemodialysis using venipuncture and a surgically created arteriovenous fistula. N Engl J Med.

[9] Ramage IJ, Bailie A, Tyerman KS, McColl JH, Pollard SG, Fitzpatrick MM (2005). Vascular access survival in children and young adults receiving long-term hemodialysis. Am J Kidney Dis.

[10] Wander JV, Moore ES, Jonasson O (1970). Internal arteriovenous fistulae for dialysis in children. J Pediatr Surg.

[11] Broyer M, Loirat C, Gagnadoux MF, Cukier J, Beurton D, Vacant J (1973). By pass and arteriovenous fistula for chronic hemodialysis in children (in French). Arch Fr Pediatr.

[12] Bourquelot P, Raynaud F, Pirozzi N (2003). Microsurgery in children for creation of arteriovenous fistulas in renal and non-renal diseases. Ther Apher Dial.

[13] Gradman WS, Lerner G, Mentser M, Rodriguez H, Kamil ES (2005). Experience with autogenous arteriovenous access for hemodialysis in children and adolescents. Ann Vasc Surg.

[14] Bagolan P, Spagnoli A, Ciprandi G, Picca S, Leozappa G, Nahom A, Trucchi A, Rizzoni G, Fabbrini G (1998). A ten-year experience of Brescia-Cimino arteriovenous fistula in children: technical evolution and refinements. J Vasc Surg.

[15] Bourquelot P, Cussenot O, Corbi P, Pillion G, Gagnadoux MF, Bensman A, Loirat C, Broyer M (1990). Microsurgical creation and follow-up of arteriovenous fistulae for chronic haemodialysis in children. Pediatr Nephrol.

[16] Garcia de Cortazar L, Gutierrez E, Delucchi MA, Cumsille MA (1999). Vascular accesses for chronic hemodialysis in children (in Spanish). Rev Med Chil.

[17] Basile C, Lomonte C, Vernaglione L, Casucci F, Antonelli M, Losurdo N (2008). The relationship between the flow of arteriovenous fistula and cardiac output in haemodialysis patients. Nephrol Dial Transplant.

[18] Butler HG, Baker LD, Johnson JM (1977). Vascular access for chronic hemodialysis: polytetrafluoroethylene (PTFE) versus bovine heterograft. Am J Surg.

[19] Brittinger WD, Walker G, Twittenhoff WD, Konrad N (1997). Vascular access for hemodialysis in children. Pediatr Nephrol.

[20] Lau KK, Jones DP, Gaber O, Nezakatgoo N (2007). Use of a “composite” vascular access graft in a young child on hemodialysis. Hemodial Int.

[21] Nghiem DD, Schulak JA, Corry RJ (1983). Management of the infected hemodialysis access grafts. Trans Am Soc Artif Intern Organs.

[22] Bennion RS, Hiatt JR, Williams RA, Wilson SE (1985). A randomized, prospective study of perioperative antimicrobial prophylaxis for vascular access surgery. J Cardiovasc Surg (Torino).

[23] Sheth RD, Brandt ML, Brewer ED, Nuchtern JG, Kale AS, Goldstein SL (2002). Permanent hemodialysis vascular access survival in children and adolescents with end-stage renal disease. Kidney Int.

[24] Chand DH, Brier M, Strife CF (2005). Comparison of vascular access type in pediatric hemodialysis patients with respect to urea clearance, anemia management, and serum albumin concentration. Am J Kidney Dis.

[25] Sheth RD, Kale AS, Brewer ED, Brandt ML, Nuchtern JG, Goldstein SL (2001). Successful use of Tesio catheters in pediatric patients receiving chronic hemodialysis. Am J Kidney Dis.

[26] Goldstein SL, Macierowski CT, Jabs K (1997). Hemodialysis catheter survival and complications in children and adolescents. Pediatr Nephrol.

[27] Schon D, Whittman D (2003). Managing the complications of long-term tunneled dialysis catheters. Semin Dial.

[28] Petersen J, Delaney JH, Brakstad MT, Rowbotham RK, Bagley CM (1999). Silicone venous access devices positioned with their tips high in the superior vena cava are more likely to malfunction. Am J Surg.

[29] Jean G, Chazot C, Vanel T, Charra B, Terrat JC, Calemard E, Laurent G (1997). Central venous catheters for haemodialysis: looking for optimal blood flow. Nephrol Dial Transplant.

[30] Saad TF (1999). Bacteremia associated with tunneled, cuffed hemodialysis catheters. Am J Kidney Dis.

[31] Beathard GA (1999). Management of bacteremia associated with tunneled-cuffed hemodialysis catheters. J Am Soc Nephrol.

[32] Maki DG, Ringer M, Alvarado CJ (1991). Prospective randomised trial of povidone-iodine, alcohol, and chlorhexidine for prevention of infection associated with central venous and arterial catheters. Lancet.

[33] Bleyer AJ (2007). Use of antimicrobial catheter lock solutions to prevent catheter-related bacteremia. Clin J Am Soc Nephrol.

[34] Valentini RP, Geary DF, Chand DH (2008). Central venous lines for chronic hemodialysis: survey of the Midwest Pediatric Nephrology Consortium. Pediatr Nephrol.

[35] Hoshal VL, Ause RG, Hoskins PA (1971). Fibrin sleeve formation on indwelling subclavian central venous catheters. Arch Surg.

[36] Gray RJ, Levitin A, Buck D, Brown LC, Sparling YH, Jablonski KA, Fessahaye A, Gupta AK (2000). Percutaneous fibrin sheath stripping versus transcatheter urokinase infusion for malfunctioning well-positioned tunneled central venous dialysis catheters: a prospective, randomized trial. J Vasc Interv Radiol.

[37] Twardowski ZJ (1998). High-dose intradialytic urokinase to restore the patency of permanent central vein hemodialysis catheters. Am J Kidney Dis.

[38] Bamgbola OF, del Rio M, Kaskel FJ, Flynn JT (2005). Recombinant tissue plasminogen activator infusion for hemodialysis catheter clearance. Pediatr Nephrol.

[39] Chand DH, Brier ME, Strife CF (2007). Effects of demographic characteristics and medical care before starting hemodialysis on vascular access in incident pediatric hemodialysis patients. Pediatr Nephrol.

[40] Davidson I, Gallieni M, Saxena R, Dolmatch B (2007). A patient centered decision making dialysis access algorithm. J Vasc Access.

[41] Turmel-Rodrigues LA, Bourquelot P, Pengloan J (2003). Hemodialysis arteriovenous fistula maturity: US evaluation. Radiology.

[42] Chand DH, Poe SA, Strife CF (2002). Venous pressure monitoring does not accurately predict access failure in children. Pediatr Nephrol.

[43] Goldstein SL, Smith CM, Currier H (2003). Noninvasive interventions to decrease hospitalization and associated costs for pediatric patients receiving hemodialysis. J Am Soc Nephrol.

[44] National Kidney Foundation (2006). KDOQI Clinical Practice Guidelines and Clinical Practice Recommendations for 2006 Updates: Hemodialysis Adequacy. Peritoneal Dialysis Adequacy, Vascular Access. Am J Kidney Dis.

